# Quantifying Early Electromechanical Integration of Cardiomyocytes Using a Minimalist PCL Nanofiber Platform

**DOI:** 10.3390/polym18010017

**Published:** 2025-12-21

**Authors:** Vitalii Dzhabrailov, Elena Turchaninova, Daria V. Kononova, Egor Ilin, Mikhail Slotvitsky, Anton Efimov, Igor Agapov, Valeriya Tsvelaya, Alexander Romanov, Konstantin Agladze

**Affiliations:** 1 E. Meshalkin National Medical Research Center of the Ministry of Health of the Russian Federation, 630055 Novosibirsk, Russia; turchaninova.ea@phystech.edu (E.T.); vts93@yandex.ru (V.T.); abromanov@mail.ru (A.R.); 2 Moscow Center for Advanced Studies, 123592 Moscow, Russia; kononova.dv@phystech.edu (D.V.K.); ilin.ed@phystech.edu (E.I.); agladze@yahoo.com (K.A.); 3 M.F. Vladimirsky Moscow Regional Clinical Research Institute, 129110 Moscow, Russia; 4 Academician V.I. Shumakov National Medical Research Center of Transplantology and Artificial Organs, Ministry of Health of the Russian Federation, 123182 Moscow, Russia; antefimov@gmail.com (A.E.); igor_agapov@mail.ru (I.A.); 5 Department of Cardiovascular Surgery (FPC and PPV), Novosibirsk State Medical University, 630091 Novosibirsk, Russia

**Keywords:** cardiac cell therapy, electrophysiological integration, polycaprolactone nanofibers, optical mapping, intercellular coupling, tissue engineering, neural network

## Abstract

A critical obstacle in cardiac cell therapy is the unpredictable and poorly understood initial electrophysiological integration of grafted cardiomyocytes into the host tissue, a process that dictates therapeutic success and arrhythmic risk. Current models fail to capture the earliest stages of functional coupling formation. Here, we employed a tailored bioengineering platform, where single cardiomyocytes were stabilized on minimalist electrospun polycaprolactone (PCL) nanofibers, to model the “graft–host” interface and study the dynamics of excitation wave transmission in real-time. Using high-speed optical mapping enhanced by a custom SUPPORT neural network, we achieved the first quantitative insights into the efficiency of nascent intercellular contacts. We determined that within the first 3 h, these initial connections are 39–44 times less effective at conducting excitation than mature contacts within the native monolayer, explaining the observed partial (46%) synchronization of grafted cells. This work provides the first direct measurement of the functional deficit during the initial minutes and hours of graft integration. It establishes that simple, inert polymer fibers can act as a catalytic scaffold to enable this fundamental biological process, offering a powerful strategy to deconstruct and ultimately control the integration of engineered tissues (or cells) for safer cell therapies.

## 1. Introduction

Myocardial regeneration represents a formidable challenge in contemporary biomedical science, with cell-based therapeutic strategies offering promising avenues for addressing ischemic cardiomyopathy and associated cardiac pathologies [1,2]. Clinical translation of cardiomyocyte transplantation remains constrained by suboptimal engraftment efficiency and proarrhythmic complications stemming from impaired electromechanical integration [3,4,5]. Current cell delivery methods, including direct intramyocardial injection and intracoronary infusion, result in substantial cell loss and poor graft retention.

The electrophysiological integration of grafted cardiomyocytes presents particularly complex challenges. Successful engraftment requires not only cell survival and structural incorporation but also establishment of functional electromechanical coupling with host tissue. The fundamental prerequisite for successful graft–host integration involves establishment of functional intercellular coupling through both gap junction-mediated and potential mechanisms. The former relies on connexin-based channels (predominantly Cx43 in ventricular myocardium) forming pathways for intercellular current flow [6,7]. The latter, termed ephaptic coupling, involves electric field interactions in nanoscale extracellular clefts without direct cytoplasmic continuity [8,9,10]. Contemporary models suggest these mechanisms may operate synergistically, with ephaptic coupling potentially providing compensatory conduction pathways during gap junction remodeling [11,12,13].

Critical to understanding graft integration dynamics is the recognition that functional maturation of intercellular connections requires a protected period of several hours post-transplantation, during which mechanical stabilization is essential for structural and electrophysiological recovery [14]. This vulnerable period is characterized by dynamic reorganization of intercellular junctions, membrane polarization establishment, and calcium handling system maturation. Previous investigation established that polymer microcarriers effectively maintain cardiomyocyte structural integrity during this vulnerable period [15], providing a stable platform for initial graft–host interaction [16].

The nanofiber carrier approach represents a significant advance in cell delivery technology [17,18]. The controlled dimensionality of these carriers allows for the creation of a suitable environment for studying the mechanisms of intercellular interaction. Another significant advantage of such carriers is that the cells attached to them are not in a suspended state. The nanofibers provide an adhesive surface for the cells in the solution. As a result, the cells do not remain spherical but instead envelop the nanofibers [15] and undergo spreading. This process allows the cells to complete a preparatory stage before forming intercellular contacts (a suspended cell cannot engage in electrophysiological interactions with other cells, making this step essential [19]).

One of the possible materials for creating biocompatible fibers is polycaprolactone (PCL). PCL nanofibers offer several functional advantages that support cardiomyocyte stabilization during the period of implantation and formation of intercellular connections. Importantly, PCL is mechanically stable, biologically inert, and sufficiently minimalist to isolate the intrinsic cellular processes involved in forming new electrical junctions. PLC fibers were also coated with fibronectin to increase cell adhesion [20]. As we see in [21], when coating PLC fibers with fibronectin, we obtain a substrate with adhesive properties comparable to a standard fibronectin-coated glass substrate. Also, to use nanofibers as cell substrates, it is necessary that the fiber be suspended rather than lying on a solid surface (otherwise, the cell will be fixed not only to the fiber, but also to the surface). This requires a scaffold made of an inert biocompatible material. The polydimethylsiloxane (PDMS) scaffold is suitable for all these conditions. It was required solely as a rigid framework to maintain the structural integrity of the entire scaffold (i.e., to support the fibers in a suspended state). The PDMS scaffold did not engage in any physiological interactions with neonatal rat cardiomyocytes and did not affect the cellular environment, as PDMS is a biologically inert material. Thus PDMS serves only as a rigid support for electrospinning and does not interact with cells. It was required solely as a rigid framework to maintain the structural integrity of the entire scaffold (i.e., to support the fibers in a suspended state). The PDMS scaffold did not engage in any physiological interactions with neonatal rat cardiomyocytes and did not affect the cellular environment (like in [22]), as PDMS is a biologically inert material [23,24].

However, there is currently no data on how cardiomyocytes initiate electrophysiological contact with each other. Thus, this investigation aims to evaluate the electrophysiological integration of carrier-stabilized cardiomyocytes (graft) with the recipient monolayer (host) during the early engraftment phase. The study will determine the time constraints for the integration of single cardiomyocytes into a confluent monolayer, as well as limitations in the propagation of excitation waves through newly formed connections to the recently integrated cardiomyocytes from the synchronized monolayer.

## 2. Materials and Methods

### 2.1. Rat Neonatal Cardiomyocyte Isolation Protocol

Rat neonatal cardiomyocytes were isolated using a standard two-day protocol adapted from the Worthington-Biochem protocol for the isolation of neonatal rat cardiomyocytes and fibroblasts (https://www.worthington-biochem.com/products/neonatal-cardiomyocyte-isolation-system/manual, accessed on 15 June 2010). Hearts were harvested from 1–4-day-old neonatal rats (*Rattus norvegicus, Sprague Dawley breed*) and immediately placed in ice-cold Ca^2+^- and Mg^2+^-free Hank’s Balanced Salt Solution (HBSS; Gibco, Oklahoma, OK, USA, 14170112). The hearts were then cleared of blood, atria, and major vessels, retaining only the ventricles.

The ventricular tissue was minced into 0.5–1 mm fragments and digested in a solution of 0.25% Trypsin-EDTA (Gibco, Oklahoma, OK, USA, 25200056) for 16 h at 4 °C. The following day, the tissue fragments were subjected to enzymatic digestion with Collagenase type II (2.25 μg/mL; Gibco, Oklahoma, OK, USA, 17101015) for 1 h at 37 °C with constant agitation. The resulting cell suspension was filtered through a 100 μm cell strainer and centrifuged at 90× *g* for 5 min.

The cell pellet was gently resuspended in Dulbecco’s Modified Eagle Medium (DMEM; Paneco, Leninskiye Gorki, Russia, C410п) by dropwise addition over a 10-min period to prevent calcium shock. To separate cardiomyocytes from fibroblasts, the cell suspension was subjected to a pre-plating step by incubation in a T75 flask (SPL Lifesciences, Busan, Korea) for 1 h in a CO_2_ incubator. After this period, the supernatant, enriched with cardiomyocytes, was collected.

The cardiomyocytes were then plated either

1. Onto 13 mm fibronectin-coated (Paneco, Leninskiye Gorki, Russia, H Fne-C) glass coverslips at a density of 100,000 cells per coverslip to obtain confluent monolayers.
2. Onto pre-conditioned polymer fiber scaffolds at a density of 50,000 cells per scaffold to obtain isolated cells on fibers.

The number of cells seeded onto the samples was determined in preliminary experiments based on the following criteria:

1. When seeding cells onto glass substrates, it is necessary to obtain a confluent monolayer to ensure that subsequent stimulation at any location yields contractions across the entire sample.
2. When seeding cells onto the polymer scaffold, it is essential to prevent electrical and mechanical coupling between cells located on fibers. This requirement ensures that stimulation of the observed cell occurs exclusively due to the monolayer cells beneath it, which form newly established intercellular contacts, rather than due to excitation transmitted from other monolayer cells, such as pacemaker cells that may accidentally set on fiber during the isolation process or cells activated by the electrode. The observed cell cannot be directly stimulated by the electrode because it is located at a considerable distance from it; however, this limitation does not apply to other cells residing on the fiber. Therefore, maintaining isolation between the cells on the fiber is critically important.

### 2.2. Preparation of PDMS Scaffolds for Electrospinning of Polymer Fibers

Polydimethylsiloxane (PDMS) (Dow Corning, Midland, MI, USA) molds served as rigid scaffolds for polymer fiber deposition. The PDMS was prepared by thoroughly mixing the two liquid components, Sylgard 184 Silicone Elastomer Base (Dow Corning, Dow Corning, Midland, MI, USA) and Sylgard 184 Curing Agent (Dow Corning, Dow Corning, Midland, MI, USA), at a 10:1 mass ratio for 10 min. The resulting mixture was poured into a 10 cm diameter Petri dish to form a layer approximately 3 mm thick. The PDMS was subsequently cured in an oven at 70 °C for 24 h. Finally, scaffolds of the desired shape were cut from the cured PDMS slab.

### 2.3. Electrospinning of Polymer Fibers onto PDMS Scaffolds

A polycaprolactone (PCL) solution was prepared by dissolving PCL powder (440744, Sigma-Aldrich, St. Louis, MO, USA) in hexafluoroisopropanol to a concentration of 15% *w*/*v*. The prepared solution was loaded into a 3 mL syringe fitted with a 20-gauge blunt-end needle and electrospun using a Nanon-01 apparatus (MECC Co., Ltd., Ogori-shi, Fukuoka, Japan). Electrospinning was performed at an applied voltage of 7 kV between the syringe needle and a grounded collector, with a solution flow rate of 0.5 mL/h maintained by a programmable Fusion 100 syringe pump. The fibers were directly deposited onto the pre-fabricated PDMS scaffolds. Subsequently, the entire construct was coated with fibronectin (Paneco, Leninskiye Gorki, Russia, H Fne-C) to enhance the adhesive properties of the polymer fibers.

### 2.4. Optical Mapping

Both cell monolayers and cells on fibers were stained using Fluo-4 AM (Lumiprobe, Moscow, Russian, 1892-500ug). A fiber scaffold with cells was then placed atop a cell monolayer, and the resulting construct was immersed in Tyrode’s salt solution (pH 7.25–7.4) for optical mapping, as previously described [25].

The fluorescence signal was recorded at a resolution of 512 × 512 pixels and a sampling rate of 34.6 frames per second using an Olympus MVX-10 Macro-View fluorescent microscope (Olympus Co., Ltd., Tokyo, Japan) equipped with a high-speed Andor iXon-3 EMCCD camera (Andor Technology Co., Ltd., Belfast, UK). A lens with a magnification of 6.3 × 1.6 was used.

Optical mapping using a confocal microscope was analyzed and processed on a Zeiss LSM 710 confocal microscope with Zen black 3.0 software (Carl Zeiss AG, Oberkochen, Germany). The signal was recorded at a resolution of 1024 × 1024 pixels. The scan time of one frame was 36 s. Thus, it took 0.74 s to scan one line. A lens with a magnification of 20 was used.

The experiment was conducted at 37 °C to maintain physiological conditions. Electrical field stimulation was applied via an inert platinum electrode. Stimulation pulses had a duration of 20 ms, an inter-pulse interval of 1000 ms, and an amplitude of 5 V. These electrical pulses were generated by a 2 MHz USB PC function generator (PCGU100, Velleman, Gavere, Belgium).

Data processing was fulfilled using the ImageJ (v1.54p) program and the associated plugins (https://imagej.net/ij/ (accessed on 5 May 2023)). ImageJ plugin (time-lapse color-coder) was used to build pseudo-3D images and activation maps.

### 2.5. SUPPORT Neural Network

Recorded optical mapping videos were denoised using a SUPPORT neural network. The training dataset comprised the very first control optical mapping recording, which was subsequently used to denoise all other recordings. The network input consisted of an image stack of 280 frames with a resolution of 512 × 512 pixels.

The same neural network was also applied to denoise confocal microscopy data. Since such data typically constitute very small stacks (10 images per stack), the stack size was artificially increased to 100 images using bilinear interpolation. This augmentation enhanced the network’s processing efficiency even with a limited amount of input data.

### 2.6. Data Processing and Statistical Analysis

Preliminary processing of optical mapping and confocal microscope data was performed using ImageJ (v1.54p). To construct activation maps, background subtraction was applied to the videos, followed by SUPPORT neural network noise reduction and Gaussian blurring. The activation map generation algorithm was implemented in Python (v3.11.13) using the following libraries: Matplotlib (v3.10.0), NumPy (v2.0.2), SciPy (v1.15.3), and Pandas (v2.2.2). The algorithm is based on increasing the glow intensity of each pixel by a certain percentage of its average brightness. This principle provides increased sensitivity of the method in comparison with the standard approach, in which a single absolute threshold is applied to all pixels.

To analyze the fluorescence intensity, plots were generated with preliminary processing in ImageJ (by using SUPPORT neural network for noise reduction), followed by data processing in Microsoft Excel and further plot generation and statistical analysis in SciDAVis (v2.7).

Spatio-temporal maps were constructed using the Stacks/Reslice function in ImageJ. To further reduce noise, a stack of spatio-temporal maps was generated within a user-defined rectangular region of interest. The height of the rectangle (in pixels) corresponded to the number of images in the resulting stack, effectively representing the spatial axis of the map. The length of the rectangle corresponded to the number of frames in the original image sequence, representing the temporal axis. Subsequent averaging of all frames along the height of the spatio-temporal map stack produced a final, significantly cleaner spatio-temporal map with reduced noise. When the longitudinal axis of the rectangle is oriented perpendicular to the wavefront (i.e., aligned with the direction of wave propagation), the tangent of the angle of the bright line (corresponding to the wavefront) in the spatio-temporal map defines the conduction velocity of the excitation wave.

## 3. Results

### 3.1. Description of the Developed Model for Studying Intercellular Contact Formation

The developed model is designed to study the rate of intercellular contact formation by seeding isolated rat neonatal cardiomyocytes onto a pre-established monolayer of the same cells. This approach enables the observation of excitation wave propagation across the monolayer and its subsequent transmission to the individual cells residing on polymer fibers.

**Figure 1:**
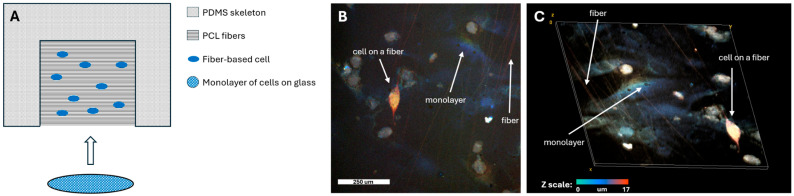
Experimental design and seeding of fiber-cultured cells onto a synchronized monolayer: (**A**) Schematic of the developed model for studying the formation of electrophysiological contacts between cells; (**B**) 2D confocal image of the cell monolayer with fibers seeded on top; (**C**) 3D confocal image of the cell monolayer with fibers seeded on top. Panels (**B**,**C**) share a common height (z-scale) color bar.

To facilitate rapid and effective electrophysiological coupling between the seeded cells and the monolayer, the cells required a solid substrate rather than being in suspension. Since the cells located on a certain substrate enter into an electromechanical interaction faster than the cells in suspension [26,27]. To this end, cells were plated on thin, biocompatible polycaprolactone (PCL) fibers. These fibers were fabricated using an electrospinning apparatus and deposited onto a scaffold made of polydimethylsiloxane (PDMS). This scaffold is necessary to maintain the fibers in a suspended state. Since the PDMS is about 3–4 mm thick, this distance is sufficient so that the fibers on it do not touch the bottom of the Petri dish. In this way, it is possible to ensure the adhesion of cells to the fiber located in the thickness of the medium. After that, such cells on the fibers can be easily transferred to the heart tissue model selected for the study. Also, the entire construct was coated with human fibronectin [21] to enhance its adhesive properties (Figure 1A).

Following a 4-day cultivation period, both the cells on the fibers and the monolayer cells were stained with the calcium-sensitive dye Fluo-4 AM, which visualizes the propagation of excitation waves within the model system. The fiber scaffolds with cells were then carefully placed directly onto the ready-made cell monolayer. At this point, the seeded cells began to integrate electrophysiologically into the existing monolayer.

Representative confocal microscopy images of the assembled model are shown in Figure 1B,C. The color scale indicates the height along the *z*-axis. The orange color of the cell on the fiber confirms its position above the monolayer, which is predominantly blue. The fibers themselves also appear orange, indicating they are located at the same height level as the seeded cell, above the monolayer.

### 3.2. Results of Optical Mapping of the Developed Model

**Figure 2:**
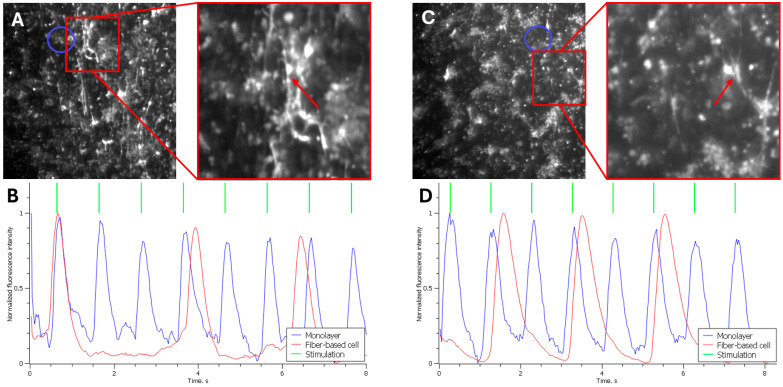
Optical mapping of fiber-cultured cells following their seeding onto a cell monolayer: (**A**) Optical mapping image of cells 15 min after seeding the fiber-cultured cells onto the monolayer. The right panel shows a magnified view of the target cell on the fiber. The blue circle on the left image indicates the region from which fluorescence intensity was measured to plot the monolayer cell activity below. (**B**) Normalized fluorescence intensity of the cells (which correlates with contractile activity, as the propagation of action potentials increases intracellular calcium concentration, triggering fluorescence). The blue trace corresponds to monolayer fluorescence, the red trace shows fluorescence of the cell on the fiber (indicated by the red arrow in the image above), and the green trace indicates the stimulation frequency applied to the monolayer via a platinum electrode (the electrode is not visible in the frame due to high magnification; it was located to the left of the shown cells, resulting in excitation wave propagation from left to right across the monolayer). (**C**) Optical mapping image of cells 176 min after seeding the fiber-cultured cells onto the monolayer. The right panel shows a magnified view of the target cell on the fiber. The blue circle on the left image indicates the region from which fluorescence intensity was measured to plot the monolayer cell activity below. (**D**) Normalized fluorescence intensity of the cells. The blue trace corresponds to monolayer fluorescence, the red trace shows fluorescence of the cell on the fiber (indicated by the red arrow in the image above), and the green trace indicates the stimulation frequency applied to the monolayer via a platinum electrode (the electrode is not visible in the frame due to high magnification; it was located to the left of the shown cells, resulting in excitation wave propagation from left to right across the monolayer).

Following the seeding of cells on fibers onto the monolayer, the propagation of excitation waves within the culture was recorded during stimulation of the monolayer via a platinum electrode. Figure 2B shows the normalized fluorescence intensity trace recorded 15 min after the experiment onset, a time point at which the cells had not yet established functional contacts.

The blue trace represents the excitation profile of the cell monolayer (mean fluorescence intensity measured near the target cell on the fiber, indicated by the blue circle in Figure 2A). As shown, the peaks of maximum fluorescence in the monolayer correspond to the 1 Hz stimulation frequency applied via the platinum electrode.

In contrast, the fluorescence trace from the cell on the fiber (red trace) shows no synchronization with the monolayer. The peaks of maximum intensity occur either randomly, coincidentally with the monolayer, or with a variable delay. This indicates that the excitation in the fiber cell is driven by its intrinsic pacemaker activity and is not yet coupled to the monolayer.

Figure 2D displays a similar fluorescence intensity trace recorded 176 min after the experiment began. By this time, the cells on the fibers had begun to establish electrophysiological coupling with the monolayer cells. The graph again shows that the fluorescence peaks of the monolayer cells (blue trace) align with the external stimulation (green trace).

The red trace represents the fluorescence intensity of the cell on the fiber (marked by the red arrow in Figure 2C). In this case, partial synchronization of the fiber cell’s activity with the monolayer is observed. Every second excitation wave is successfully transmitted from the monolayer to the single cell. A significant delay in excitation transmission to the fiber cell is also evident, which we attribute to an insufficient number of established intercellular contacts.

Several fiber cells exhibiting a similar level of synchronization with the monolayer were identified across multiple experiments after approximately 3 h. Out of 42 waves propagating through the monolayer, these cells fired synchronously (with a similar transmission delay as described) in 20 instances. Thus, the excitation wave was transmitted from the monolayer to the seeded cells in 46% of cases. This indicates successful, yet incomplete, integration of the cells into the monolayer, with nearly every second wave propagating to the cells on the fibers.

### 3.3. Evaluation of the Pre-Trained Neural Network for Noise Removal

**Figure 3:**
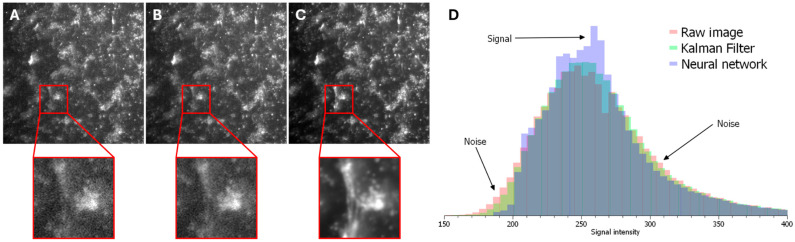
Performance evaluation of the developed neural network for noise removal and signal-to-noise ratio determination: (**A**) Top: Frame from a raw optical mapping recording. Bottom: Magnified view of the region within the red square. (**B**) Top: Frame from an optical mapping recording processed with a Kalman filter. Bottom: Magnified view of the region within the red square. (**C**) Top: Frame from an optical mapping recording processed with the developed neural network. Bottom: Magnified view of the region within the red square. (**D**) Intensity histogram of frames (**A**–**C**), indicating noise and signal regions.

To ensure the highest accuracy in measurements, it is essential to remove extraneous noise from optical mapping recordings, as noise can significantly distort the results. Figure 3 shows frames from optical mapping videos: Figure 3A displays the raw video, Figure 3B shows the result after applying a Kalman filter, and Figure 3C demonstrates the output after processing with the SUPPORT neural network.

As evident in the magnified insets, the application of the neural network substantially reduces noise levels. This improvement is further quantified in Figure 3D. The signal intensity (range 230–280 arbitrary units) increases progressively in the sequence: raw image → Kalman filter → neural network. Conversely, the noise intensity (ranges 150–230 and 280–400 arbitrary units) decreases in the same sequence.

Quantitative assessment of the signal-to-noise ratio (SNR) yielded the following values: 0.61 for the raw image, 1.70 for the Kalman filter, and 3.55 for the neural network. These results demonstrate that the implemented neural network significantly enhances the SNR, thereby facilitating data analysis and improving the accuracy of subsequent measurements.

### 3.4. Evaluation of Excitation Wave Transmission Time Between Monolayer Cells and Through Newly Formed Intercellular Contacts

As demonstrated in Section 3.2, a significant delay exists in the transmission of excitation waves from the monolayer to cells on fibers. Since the formation of electrophysiological coupling between cells requires time, the speed of intercellular wave transmission is expected to increase over time (toward its normal value). Consequently, as the density of intercellular contacts increases, the efficiency of excitation wave transmission to the seeded cells improves. For simplicity, the term “intercellular contacts” is used here, though this may include both gap junctions and ephaptic coupling. Thus, the inverse of the wave transmission time across these contacts serves as a reasonable measure of transmission efficiency.

**Figure 4:**
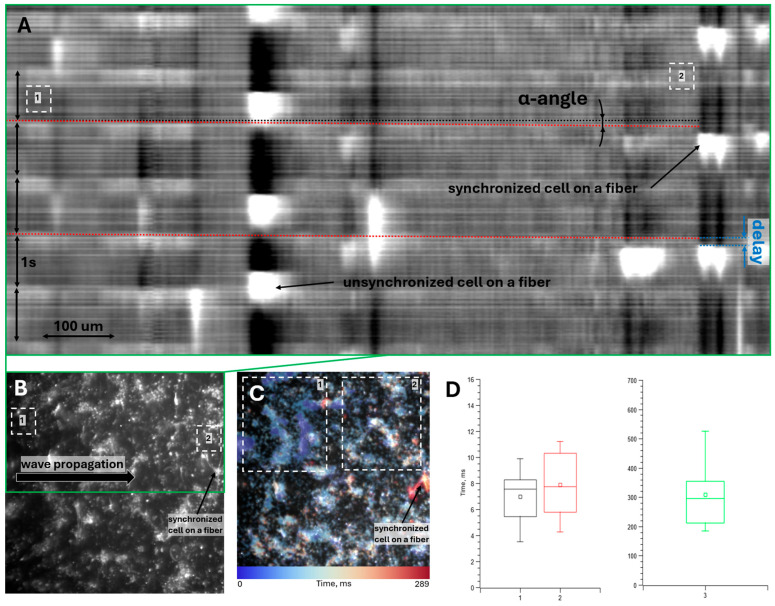
Determination of excitation wave propagation velocity in the monolayer and its transmission time to the fiber-cultured cell: (**A**) Averaged (across the green rectangle in (**B**)) spatio-temporal map (kymograph) of fluorescence intensity. Red dashed lines indicate excitation wave propagation across the monolayer. The black dashed line is an isochrone showing the slope of the red dashed line (1/tan(α-angle) corresponds to the wave propagation velocity through the monolayer). The blue marking indicates the delay in excitation wave transmission from the monolayer to the fiber cell. Regions 1 and 2 represent the initiation and termination points of wave propagation through the monolayer, respectively. (**B**) Optical mapping result of the monolayer with seeded fiber-cultured cells. The green rectangle marks the region used for averaging the spatio-temporal map above. Regions 1 and 2 indicate the initiation and termination points of wave propagation through the monolayer, respectively (the stimulating platinum electrode is not visible in the frame due to high magnification but was positioned to the left of the shown cells, resulting in left-to-right wave propagation as indicated by the arrow). (**C**) Activation map of excitation wave propagation for this optical mapping experiment. Regions 1 and 2 are marked to demonstrate the color difference in the activation map between these regions and to determine the direction of wave propagation through the monolayer. (**D**) Transmission times of excitation waves: (1) through intercellular contacts within the monolayer (cell count determined from optical mapping videos), (2) through intercellular contacts within the monolayer (cell count estimated from distance between Regions 1 and 2 in (B) and average monolayer cell size), and (3) through newly formed intercellular contacts between monolayer cells and the fiber-cultured cell.

Figure 4A shows the averaged spatio-temporal map of cell fluorescence intensity along the wave propagation path (illustrated in Figure 4B). The α-angle marked in Figure 4A reflects the conduction velocity of the excitation wave across the monolayer. This angle is relatively small due to the inherent trade-off between spatial and temporal resolution; the wave propagates across the entire field of view within 2–3 frames. Nevertheless, with sufficient data, the wave conduction velocity can be estimated. The blue marking in Figure 4A indicates the delay in wave transmission from the monolayer to the fiber cell, averaging approximately 10 frames (more precise calculations are provided below).

Figure 4C presents an activation map of excitation wave propagation across the monolayer. Region 1 appears brighter blue than Region 2, confirming left-to-right wave propagation (as shown in Figure 4D). The target cell on the fiber is marked in orange, indicating significantly delayed activation compared to adjacent monolayer cells.

Supplementary Video S1 shows the real-time propagation of an excitation wave across the monolayer and its subsequent transmission to the cell on the fiber. The video panel on the left displays the original recording (subject only to noise removal), while the panel on the right shows the background-subtracted version to enhance the visualization of the fluorescence increase in the cells.

Due to the small α-angle and brief overall propagation time, which are challenging to measure accurately, an alternative method was employed: the time difference between fluorescence peaks in Region 1 (wave initiation) and Region 2 (wave termination near the fiber cell) in Figure 4A,B was calculated. We model action potential propagation along a cell chain, assuming the intercellular transmission time dominates the intracellular conduction time. Thus, knowing the number of cells traversed by the wave allows estimation of the intercellular transmission time within the monolayer. This can then be compared to the wave transmission time from the monolayer to the fiber cell, which solely depends on trans-contact propagation.

Figure 4D shows the calculated average excitation wave transmission times across intercellular contacts. Plots 1 and 2 represent the estimated intercellular transmission time within the monolayer. Since cell boundaries are indistinct in optical mapping videos, two methods were used: Plot 1—direct cell counting from videos; Plot 2—estimating cell count based on the distance between Regions 1 and 2 (Figure 4B) and the average spread cell size in the monolayer (100 μm). The results from both methods agree within error margins. Plot 3 in Figure 4D shows the transmission time across newly formed contacts between the fiber cell and the monolayer, which significantly exceeds the intracellular transmission time within the monolayer.

The efficiency ratio of newly formed contacts (over 3 h) to monolayer contacts can thus be estimated as the inverse ratio of the transmission times shown in Figure 4D. The results were

(44 ± 11)—using optical mapping-based cell counting;

(39 ± 10)—using distance and average cell size-based estimation.

To confirm the statistical significance of the results, an analysis described in Appendix A was performed. This analysis indicates that the probability of a synchronized cell on the fiber activating randomly, independently of the monolayer’s activation, is close to zero (0.06%).

**Figure 5:**
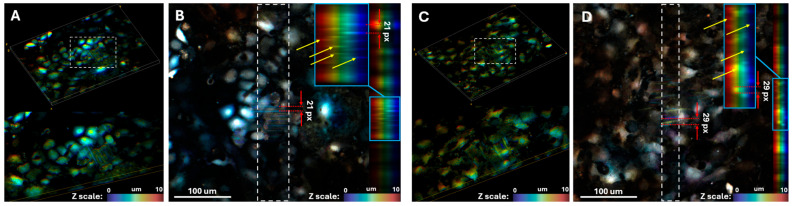
Application of confocal microscopy-based optical mapping: (**A**) Top: 3D confocal image of a cell monolayer. Bottom: Magnified view of the region indicated by the white rectangle above. (**B**) Confocal image of a cell monolayer (top view). Right: Averaged side-projection of the image, with the averaging region marked by a white rectangle. The area within the blue rectangle shows a magnified view of the side projection. Yellow arrows indicate bands of increased fluorescence intensity resulting from confocal scanning of fluorescing cells (conducting excitation waves). Red arrows mark the distance between two adjacent bands (in pixels), used to determine the monolayer contraction period. (**C**) Top: 3D confocal image of a cell monolayer. Bottom: Magnified view of the region indicated by the white rectangle above. (**D**) Confocal image of a cell monolayer (top view). Right: Averaged side-projection of the image, with the averaging region marked by a white rectangle. The area within the blue rectangle shows a magnified view of the side projection. Yellow arrows indicate bands of increased fluorescence intensity resulting from confocal scanning of fluorescing cells (exhibiting intracellular calcium dynamics). Red arrows mark the width of a single band (in pixels), used to determine the period of intracellular calcium dynamics.

To further investigate the formation of electrophysiological contacts between cells, we developed a confocal microscopy-based optical mapping approach. Following staining with a calcium-sensitive dye, the cell monolayer is scanned using a confocal microscope. As the scanning proceeds layer-by-layer from top to bottom, cells fluoresce multiple times during a single frame acquisition, resulting in distinct bright bands in the final image. Figure 5A shows a spontaneously contracting cluster of rat neonatal cardiomyocytes. This experiment shows a proof of concept for using optical mapping on a confocal microscope.

Knowing the confocal microscope scanning speed enables calculation of the time interval between successive excitation waves in the monolayer. In Figure 5B, the 21-pixel distance between adjacent bands (red arrows), combined with the image resolution (1024 × 1024 pixels) and frame acquisition time (36 s), yields an excitation wave period of 0.74 s in the monolayer. This approach thus provides 3D optical mapping data with both high spatial and temporal resolution.

Another advantage of this method is its capacity for 3D mapping. In Figure 5B, yellow arrows indicate bands corresponding to excitation waves at different depths, demonstrating the potential for studying excitation wave synchronization across different cell layers. By stimulating the bottom cell layer at a fixed frequency, we can investigate how and when excitation waves propagate to upper cell layers—in this case, single cells on polymer fibers.

This method also enables investigation of intracellular calcium dynamics. Figure 5C,D show curved rather than straight fluorescence patterns, indicating longer intracellular fluorescence duration compared to wave propagation. This measured duration is 1.0 s (29 pixels on Figure 5D).

## 4. Discussion

In this study, we developed and validated a novel experimental model that leverages advanced polymer materials to dissect the early stages of electrophysiological integration in cardiac tissue engineering. The core of our approach was a minimalist scaffold made from PCL nanofibers on a PDMS frame, which provided critical mechanical stabilization for single cardiomyocytes during the vulnerable period following their grafting onto a host monolayer.

Our findings yield two major conclusions. First, we successfully quantified, in real time, the formation of the minimal functional intercellular units required for excitation wave transmission. The calculated 39–44-fold lower efficiency of these nascent contacts compared to mature monolayer junctions provides a fundamental, previously unmeasured benchmark for the early phase of graft-host integration. This quantitative insight is vital for predicting and mitigating arrhythmogenic risks in cell-based therapies [28].

Second, and most significantly from a materials science perspective, we observed a paradoxical “catalytic” function of the biologically inert PCL fibers. By offering a simple, minimalistic, and sufficient physical support, the fibers enabled the cardiomyocytes to autonomously establish functional electromechanical couplings without the need for a complex, naturally mimetic substrate. This underscores a key engineering principle: rather than attempting to fully replicate the native extracellular matrix, a minimalist synthetic polymer scaffold can be sufficient to catalyze and support the innate biological capacity of cells for self-organization and integration. This work establishes a robust polymer-based platform for future investigations into the fundamental mechanisms of intercellular communication and for the preclinical optimization of engineered tissue constructs.

A method of optical mapping using a confocal microscope was also presented. This method combines high spatial (since it refers to scanning microscopy methods) and temporal (since due to fluorescence it becomes possible to interpret temporal data using spatial data) resolution simultaneously. The proposed method, however, is subject to several constraints. A critical requirement for advancing the study of cell electromechanical integration is the precise synchronization between the stimulation period of the underlying monolayer (t_1_) and the time required to scan a single frame (t_2_). When t_2_ is an integer multiple of t_1_, the 3D model (Figure 5A) displays well-defined, vertically aligned bands, which facilitates a clear evaluation of integration efficacy in the stimulated monolayer and the adjacent upper layer. In contrast, a lack of synchrony (where t_2_ is not a multiple of t_1_) results in a disordered band pattern, making it impossible to establish a correlation between the calcium activity of the bottom and top layers. An additional limitation concerns the *z*-axis resolution, which is restricted by the allowable stimulation duration. Extended electrical exposure leads to monolayer damage, thus imposing a limit on the number of scannable layers and the achievable resolution along the *z*-axis. Nevertheless, this optical mapping approach demonstrates significantly higher precision than standard methodologies. For future studies of cardiomyocyte integration into confluent monolayers, this confocal optical mapping method will be employed to achieve enhanced spatial and temporal resolution.

This study has several limitations. The quantitative assessment of contact efficiency was derived from 2D optical mapping data. While the SUPPORT neural network significantly enhanced signal quality, the complex three-dimensional nature of the interaction between the grafted cell on the fiber and the host monolayer may introduce variables not fully captured in a 2D projection. Furthermore, while we established the formation of functional electrical coupling, the specific contributions of gap junctional versus ephaptic mechanisms to these nascent contacts remain to be elucidated. However, the confocal optical mapping methodology we have begun to develop and present here offers a direct pathway to address these limitations in future work. This 3D approach will allow for precise spatial resolution of excitation wave propagation across different Z-planes, enabling us to validate the current model and dissect the mechanistic underpinnings of the integration process with greater fidelity.

## 5. Conclusions

In conclusion, by employing a minimalist polycaprolactone nanofiber platform, we have provided the first direct quantitative assessment of the initial functional integration between grafted and host cardiomyocytes. Our key finding is that nascent intercellular contacts formed within the first hours are 39–44 times less efficient in conducting excitation than mature counterparts, establishing a fundamental benchmark for this critical period. This work underscores that simple polymer scaffolds, by providing essential mechanical stabilization, can act as catalytic enablers of biological processes, thereby offering a powerful and necessary tool to deconstruct the mechanisms of cardiac tissue engineering for safer therapeutic applications.

## Figures and Tables

**Figure A1:**
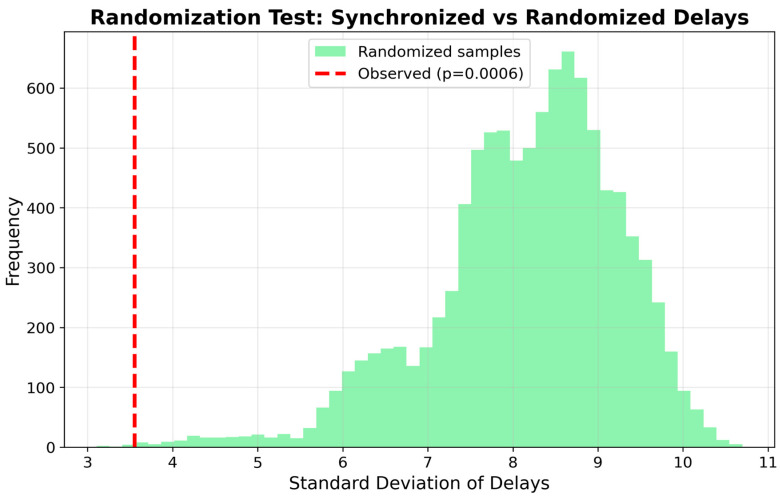
Assessment of non-random activation timing for integrated cells. The green histogram depicts the null distribution of standard deviations generated by Monte Carlo resampling (10,000 iterations, sample size m = 20) from the combined dataset of all delay values (M = 38). The observed standard deviation for synchronized cells is indicated by the red dashed line.

## Data Availability

Raw data (optical mapping and confocal microscopy) could be found at Data Repository: https://doi.org/10.5281/zenodo.17497440.

## Supplementary Materials

The following supporting information can be downloaded at: https://www.mdpi.com/article/10.3390/polym18010017/s1, Video S1: The real-time propagation of an excitation wave across the monolayer and its subsequent transmission to the cell on the fiber.

## Abbreviations

The following abbreviations are used in this manuscript:

PDMS	Polydimethylsiloxane
PCL	Polycaprolactone

## Appendix A

To assess the statistical significance of the obtained results, a statistical analysis was performed. The analysis was designed as follows: to compare the difference in the activation time of synchronized cells (which we consider to have entered an electromechanical interaction with the monolayer) and the activation time of adjacent monolayer cells with the difference in the activation time of non-synchronized cells (which we consider not to have entered an electromechanical interaction with the monolayer) and the activation time of adjacent monolayer cells.

In the experiment, for synchronized cells, *n* = 5 biological replicates were performed, during which m = 20 episodes of activity synchronous with the monolayer were recorded (i.e., the cell activates after a certain delay (approximately 10 frames) following monolayer activation, as shown in Figure 2D). For non-synchronized cells (whose activity we consider independent of the adjacent monolayer cells, as shown in Figure 2B), *n* = 9 biological replicates were performed, yielding l = 18 recordings of their activity.

Subsequently, the time interval between cell activation and the closest occurrence of wave propagation through the monolayer was determined for all M = 20 + 18 = 38 recorded events. Analysis of these delay times reveals that the delays for all synchronized cells are exclusively positive with a small deviation: (7.9 ± 3.6) frames. In contrast, for non-synchronized cells, the mean delay can be stated as zero within the margin of error: (3.4 ± 11.0) frames.

To test the hypothesis that “the activation of synchronized cells occurs after a strictly defined time following monolayer activation, whereas the activation of non-synchronized cells is independent of monolayer activation,” the following test was simulated. From the total set of M = 38 calculated delay values, a random sample of m = 20 values was drawn, and its standard deviation was computed. This procedure was repeated 10,000 times, and a histogram of these standard deviations was constructed (Figure A1).

The hypothesis under test can be reformulated as follows: the delays of synchronized cells are constant, while the delays of non-synchronized cells are random. Therefore, under our conditions, the probability of rejecting this hypothesis (the *p*-value) can be calculated as the proportion of cases in which the standard deviation of a random sample is less than the standard deviation observed for the real sample of synchronized cell delays. In this case, the probability is *p*-value = 0.0006.

Thus, we can conclude that there is very high statistical significance for the fact that the activation of synchronized cells depends on monolayer activation, unlike the activation of non-synchronized cells.

## References

[1] Zou Y., Li L., Li Y., Chen S., Xie X., Jin X., Wang X., Ma C., Fan G., Wang W. (2021). Restoring cardiac functions after myocardial infarction-ischemia/reperfusion via an exosome anchoring conductive hydrogel. ACS Appl. Mater. Interfaces.

[2] George J.C., Goldberg J., Joseph M., Abdulhameed N., Crist J., DAS H., Pompili V.J. (2008). Transvenous intramyocardial cellular delivery increases retention in comparison to intracoronary delivery in a porcine model of acute myocardial infarction. J. Interv. Cardiol..

[3] Yu J.K., Liang J.A., Weinberg S.H., Trayanova N.A. (2022). Computational modeling of aberrant electrical activity following remuscularization with intramyocardially injected pluripotent stem cell-derived cardiomyocytes. J. Mol. Cell. Cardiol..

[4] Chen H.S.V., Kim C., Mercola M. (2009). Electrophysiological Challenges of Cell-Based Myocardial Repair. Circulation.

[5] Mayourian J., Savizky R.M., Sobie E.A., Costa K.D. (2016). Modeling electrophysiological coupling and fusion between human mesenchymal stem cells and cardiomyocytes. PLoS Comput. Biol..

[6] Severs N.J., Bruce A.F., Dupont E., Rothery S. (2008). Remodelling of gap junctions and connexin expression in diseased myocardium. Cardiovasc. Res..

[7] Agladze N.N., Halaidych O.V., Tsvelaya V.A., Bruegmann T., Kilgus C., Sasse P., Agladze K.I. (2017). Synchronization of excitable cardiac cultures of different origin. Biomater. Sci..

[8] Lin J., Abraham A., George S.A., Greer-Short A., Blair G.A., Moreno A., Alber B.R., Kay M.W., Poelzing S. (2022). Ephaptic Coupling Is a Mechanism of Conduction Reserve During Reduced Gap Junction Coupling. Front. Physiol..

[9] Lin J., Keener J.P. (2013). Ephaptic coupling in cardiac myocytes. IEEE Trans. Biomed. Eng..

[10] Gourdie R.G. (2019). The cardiac gap junction has discrete functions in electrotonic and ephaptic coupling: Gap junction function in cardiac conduction. Anat. Rec..

[11] Haraguchi Y., Shimizu T., Yamato M., Kikuchi A., Okano T. (2006). Electrical coupling of cardiomyocyte sheets occurs rapidly via functional gap junction formation. Biomaterials.

[12] Veeraraghavan R., Lin J., Hoeker G.S., Keener J.P., Gourdie R.G., Poelzing S. (2015). Sodium channels in the Cx43 gap junction perinexus may constitute a cardiac ephapse: An experimental and modeling study. Pflugers Arch..

[13] Kucera J.P., Rohr S., Rudy Y. (2002). Localization of sodium channels in intercalated disks modulates cardiac conduction. Circ. Res..

[14] Slotvitsky M.M., Tsvelaya V.A., Podgurskaya A.D., Agladze K.I. (2020). Formation of an electrical coupling between differentiating cardiomyocytes. Sci. Rep..

[15] Balashov V., Efimov A., Agapova O., Pogorelov A., Agapov I., Agladze K. (2018). High resolution 3D microscopy study of cardiomyocytes on polymer scaffold nanofibers reveals formation of unusual sheathed structure. Acta Biomater..

[16] Aitova A., Scherbina S., Berezhnoy A., Slotvitsky M., Tsvelaya V., Sergeeva T., Turchaninova E., Rybkina E., Bakumenko S., Sidorov I. (2023). Novel molecular vehicle-based approach for cardiac cell transplantation leads to rapid electromechanical graft-host coupling. Int. J. Mol. Sci..

[17] Pina S., Ribeiro V.P., Marques C.F., Maia F.R., Silva T.H., Reis R.L., Oliveira J.M. (2019). Scaffolding strategies for tissue engineering and regenerative medicine applications. Materials.

[18] Li W.J., Laurencin C.T., Caterson E.J., Tuan R.S., Ko F.K. (2002). Electrospun nanofibrous structure: A novel scaffold for tissue engineering. J. Biomed. Mater. Res..

[19] Folkman J., Moscona A. (1978). Role of cell shape in growth control. Nature.

[20] Kumar N., Sridharan D., Palaniappan A., Dougherty J.A., Czirok A., Isai D.G., Mergaye M., Angelos M.G., Powell H.M., Khan M. (2020). Scalable biomimetic coaxial aligned nanofiber cardiac patch: A potential model for “clinical trials in a dish”. Front. Bioeng. Biotechnol..

[21] Teplenin A., Krasheninnikova A., Agladze N., Sidoruk K., Agapova O., Agapov I., Bogush V., Agladze K. (2015). Functional analysis of the engineered cardiac tissue grown on recombinant spidroin fiber meshes. PLoS ONE.

[22] Slotvitsky M., Berezhnoy A., Scherbina S., Rimskaya B., Tsvelaya V., Balashov V., Efimov A.E., Agapov I., Agladze K. (2022). Polymer kernels as compact carriers for suspended cardiomyocytes. Micromachines.

[23] van Meer B.J., de Vries H., Firth K.S.A., van Weerd J., Tertoolen L.G.J., Karperien H.B.J., Jonkheijm P., Denning C., Ijzerman A., Mummery C. (2017). Small molecule absorption by PDMS in the context of drug response bioassays. Biochem. Biophys. Res. Commun..

[24] Tanyeri M., Tay S. (2018). Viable cell culture in PDMS-based microfluidic devices. Methods Cell Biol..

[25] Kudryashova N., Tsvelaya V., Agladze K., Panfilov A. (2017). Virtual cardiac monolayers for electrical wave propagation. Sci. Rep..

[26] Bizy A., Klos M. (2020). Optimizing the use of iPSC-CMs for cardiac regeneration in animal models. Animals.

[27] Cui H., Liu C., Esworthy T., Huang Y., Yu Z.X., Zhou X., San H., Lee S.-J., Hann S.Y., Boehm M. (2020). 4D physiologically adaptable cardiac patch: A 4-month in vivo study for the treatment of myocardial infarction. Sci. Adv..

[28] Chong J.J.H., Yang X., Don C.W., Minami E., Liu Y.W., Weyers J.J., Mahoney W.M., Van Biber B., Cook S.M., Palpant N.J. (2014). Human embryonic-stem-cell-derived cardiomyocytes regenerate non-human primate hearts. Nature.

